# What Are the Effects of Moso Bamboo Expansion into Japanese Cedar on Arbuscular Mycorrhizal Fungi: Altering the Community Composition Rather than the Diversity

**DOI:** 10.3390/jof9020273

**Published:** 2023-02-18

**Authors:** Guiwu Zou, Binsheng Wu, Baodong Chen, Yaying Yang, Yan Feng, Jiahui Huang, Yuanqiu Liu, Philip J. Murray, Wei Liu

**Affiliations:** 1 Jiangxi Provincial Key Laboratory of Silviculture, Jiangxi Agricultural University, Nanchang 330045, China; 2 School of Art and Landscape, College of Forestry, Jiangxi Agricultural University, Nanchang 330045, China; 3 Positioning Observation Station of Forest Ecosystem in Lushan, Jiujiang 332000, China; 4 Research Center for Eco-Environmental Sciences, Chinese Academy of Sciences, Beijing 100085, China; 5 University of Chinese Academy of Sciences, Beijing 100049, China; 6 Administration of Lushan Natural Reserve, Jiujiang 332000, China; 7 School of Agriculture, Food and Environment, Royal Agricultural University, Cirencester GL7 6JS, UK

**Keywords:** AMF response, bamboo invasion, *Cryptomeria japonica*, high-throughput sequencing, *Phyllostachys edulis*

## Abstract

The unbridled expansion of moso bamboo (*Phyllostachys edulis*) occurs throughout the world and has a series of consequences. However, the effect of bamboo expansion on arbuscular mycorrhizal fungi (AMF) is still poorly understood. We assessed the changes in the AMF community during bamboo expansion into Japanese cedar (*Cryptomeria japonica*) forests by analyzing AMF in three forest types—Japanese cedar (JC), bamboo-cedar mixed (BC) and moso bamboo (MB)—using 454 pyrosequencing technology. We found that the AMF community composition differed significantly among forest types. The relative abundance of *Glomerales* decreased from 74.0% in JC to 61.8% in BC and 42.5% in MB, whereas the relative abundance of *Rhizophagus* increased from 24.9% in JC to 35.9% in BC and 56.7% in MB. Further analysis showed that soil characteristics explained only 19.2% of the AMF community variation among forest types. Hence, vegetation is presumably the main driver of the alteration of the AMF community. The *α* diversity of AMF was similar between JC and MB, although it was higher in BC. Overall, this research sheds more light on AMF community dynamics during moso bamboo expansion. Our results highlight that the consequences of bamboo expansion in monoculture forests differ from those in mixed forests.

## 1. Introduction

Plant invasion is the most important driver of biodiversity decline, threatening ecological and socioeconomic systems worldwide [1,2]. The term “invasive plant” generally refers to exotic species, but native species can also become invasive when their expansion is unbridled. Moso bamboo (*Phyllostachys edulis*), a tree-like bamboo species, is a typical native invasive plant in Asia [3]. Moso bamboo is an important forest resource due to its versatility, serving as a source of timber and food and as an ornament [4]. Moreover, recent research showed that moso bamboo forests are also important carbon sinks [5]. Meanwhile, the rapid expansion of this species has caused several problems [4], drastically changing forest structures [6], decreasing biodiversity [7,8], causing the stagnation of community succession [9], and reducing carbon storage [10]. Furthermore, biogeochemical cycles have also been considerably altered by bamboo expansion. For example, bamboo expansion could accelerate the biogeochemical Si cycle [11], stimulate litter decomposition [12], and increase soil available P [13].

The belowground ecosystem is also closely correlated with the vegetation and soil biochemical cycles [14]; hence, bamboo expansion inevitably altered the belowground ecosystems. Our previous research revealed that soil fauna groups with low nutrient levels were vulnerable when confronted with bamboo expansion [15]. Liu et al. [16] reported that the effects of bamboo expansion on soil microbial composition depended on site and forest type. The richness of bacteria and the *α* diversity of fungi have been found to increase after bamboo expansion [17,18].

Arbuscular mycorrhizal fungi (AMF) are obligate symbionts of more than 80% of vascular plant species [19]. They can benefit plant growth by providing the plant with nutrients and improving soil structure both under normal and stress conditions [20,21,22]. Additionally, AMF boosted plant growth by increasing the content of leaf chlorophyll and amino acid and alleviating salinity stress by reducing H_2_O_2_ and malondialdehyde levels, as well as increasing antioxidant-related enzyme activities [23]. AMF can also reduce the accumulation of heavy metals (beryllium) and mitigated the damage of heavy metals by the upregulation of osmoprotectants [24]. Further study revealed that AMF improves plant growth by diminishing the inhibitory effect of drought stress on cell development and by shortening the cycle of cell division [25]. Due to the various effects of AMF on plants, plant invasion success is consequently related to enhanced plant–arbuscular mycorrhizal fungi associations with introduced plants [26].

Although Qin et al. [27] revealed that the expansion of moso bamboo into a broad-leaved mixed forest increased AMF biomass but decreased AMF diversity, our knowledge about the effects of bamboo expansion on AMF remains limited due to challenges related to methods (Qin et al. used phospholipid-derived fatty acids to identify taxa). In addition, changes in the microorganism community during bamboo expansion depend on the invaded forest type [16], and the AMF are intimately related to plant diversity [28]. Hence, the alteration of AMF during bamboo expansion into monoculture forest is likely to be different from that which occurs under the scenario of bamboo expanding into mixed forests. Moreover, monoculture forest is a good subject for studying the influence of bamboo expansion on the AMF community because uncertainty can be reduced. However, the bamboo expansion-induced change of AMF in monoculture forests remains unclear. Due to the facilitation of plant growth by AMF, ascertaining the response of AMF community potentially provides new perspectives on the control of bamboo expansion.

To reveal the effects of bamboo expansion on AMF in monoculture forests, we used the 454 high-throughput sequencing technology to track the AMF community in different vegetation types during the expansion of bamboo into Japanese cedar (*Cryptomeria japonica*) forest at Lushan Mountain Nature Reserve, China. It has been well demonstrated that AMF diversity is positively related to plant diversity [28,29], and that AMF are obligately dependent on host plants [30]. Hence, we hypothesized that (1) the mixed forest formed by bamboo expansion has the highest AMF diversity and (2) the AMF community composition in bamboo forest would be different from that in Japanese cedar forest.

## 2. Materials and Methods

### 2.1. Study Site Description

The study site was located in Lushan National Nature Reserve, city of Jiujiang, Jiangxi Province (29°26′−29°41′ N, 115°52′−116°08′ E). It has a subtropical humid monsoon climate. The annual average temperature of Lushan is 11.4°C, with maximum and minimum temperatures of 32.8°C and −16.8°C, respectively. The annual average precipitation is 1929 mm and is mainly concentrated from April to July, accounting for approximately 70% of the annual precipitation [31]. The soil types include mountain red loam, yellow loam, yellow-brown loam, brown loam, and mountain meadow soil. The soil of the experimental site in this study is yellow-brown loam.

On the basis of a comprehensive survey, a Japanese cedar stand with typical bamboo expansion was selected at Jinzhuping (29°32′45.36″ N, 115°57′19.62″ E, 964 m). At the interface of the bamboo expansion into the Japanese cedar area, we established three 30 m × 30 m plots in the Japanese cedar forest (JC), bamboo-cedar mixed forest (BC), and moso bamboo forest (MB), in October 2014. In the JC plots, there was a sparse understory flora consisting of *Lindera reflexa*, *Symplocos stellaris*, *Callicarpa cathayana*, *Lophatherum gracile*, *Cyclosorus parasiticus*, and *Woodwardia japonica*. The understory in MB was also sparse and dotted with the following species: *Hydrangea chinensis*, *Camellia sinensis*, *Ardisia crenata*, *Cyclosorus parasiticus*, and *Liriope spicata*. In the BC, there was an understory of *Camellia sinensis*, *Lindera reflexa*, *Corylopsis sinensis*, *Platycarya strobilacea*, *Rhododendron ovatum*, *Tricyrtis macropoda*, *Acer palmatum*, *Padus buergeriana*, *Photinia villosa*, *Goodyera schlechtendaliana*, and *Cyclosorus paresiticus*. Overall, the understory coverage was very low in the three stands.

### 2.2. Soil Sampling and the Determination of Chemical Characteristics

Soil was collected from nine points in each plot and mixed into one sample, according to two separate layers (0−10 cm and 10−20 cm), considering that AM hyphae and total fungal colonization may differ between these layers [32]. A total of 18 soil samples were collected (3 forest types × 2 layers × 3 replicates). All samples were placed into sealed plastic bags and stored in an icebox until they were transported to the laboratory. In the laboratory, each sample was divided into two subsamples after being mixed and sifted through a 2 mm sieve. One subsample was air-dried and then ground to pass through a 0.149 mm mesh for future chemical characteristic analyses, and another subsample was stored at −80°C for subsequent DNA extraction.

Soil pH was determined by glass electrode under the condition of 1:2 (*w*/*v*) mixture of soil and water. The content of soil organic matter (SOM) was determined by the dichromate oxidation method [33]. Total phosphorus (TP) was determined by the molybdenum blue colorimetric method after oxidation by H_2_SO_4_-HClO_4_ and extracted with 0.5 mol L^−1^ sodium bicarbonate (NaHCO_3_), respectively. The total nitrogen (TN) content was determined by Kjeldahl method [33]. The available potassium (AK) was determined using the flame photometry method after extracted with 1.0 M ammonium acetate (pH 7.0).

### 2.3. DNA Extraction, PCR Amplification and Molecular Identification of AMF

Soil DNA was extracted from 0.5 g fresh soil samples using the FastDNA SPIN Kit for soil (Aidlab Biotechnologies Co., Ltd., Beijing, China) according to the manufacturer’s instructions. The extracted soil DNA samples were diluted to 10 ng μL^−1^ with double-distilled H_2_O and stored at −20 °C for subsequent molecular analysis.

A nested polymerase chain reaction (PCR) was used to overcome the difficulty of amplification caused by the presence of interfering substances such as humic acid in soil. The AML1/AML2 primers (for detailed sequences 5′–3′, see [34]) were used in the first PCR reaction and the NS31/AM1 (for detailed sequences 5′–3′, see [35,36]) were used in the second PCR. The PCR system and conditions were adapted from Xiang et al. [37], and the detailed conditions of the experiment are described by Zou et al. [29].

The sequences of quantified amplicons were determined on a 454-PLX+ system (Shanghai, China). Overall, the average number of sequencing data per sample was 10,000 and the average length of the sequence read length was 300−600 bp. The availed sequences (ambiguous nucleotides were discarded) were clustered into operational taxonomic units (OTUs) according to the 97% identity threshold, using the unsupervised Bayesian clustering algorithm CROP. Simultaneously, the most abundant sequence in each OTU was selected as the representative sequence. The sequences were clustered by using Usearch (version 7.1 http://drive5.com/uparse/, accessed on 27 November 2015). The fungal taxonomy was identified with UNITE (version 6.0 http://unite.ut.ee/index.php, accessed on 27 November 2015) [38]. The OTUs that could not be identified at the family or class taxon levels were verified with the National Center for Biotechnnology Information Genbank (http://www.ncbi.nlm.nih.gov/, accessed on 27 November 2015).

### 2.4. Statistical Analyses

After the data were tested for normality and homogeneity, one-way ANOVA was used to determine the differences in soil chemical properties, AM fungal community diversity indexes and the relative abundance of individual taxa among forest types. Multiple comparisons were carried out using the least significant difference (LSD) method for variables with significant differences, and the results of multiple comparisons were corrected by the Benjamini–Hochberg (BH) method. The overall pattern of AMF in different forest types was analyzed by principal component analysis (PCA) after the OTU data were Hellinger transformed. The significance of overall community differences was further determined by non-parametric multivariate analysis of variance (PERMANOVA). Redundancy analysis (RDA) was performed to reveal the correlations between soil chemical characteristics and the AMF community. The significance of each axis and soil chemical characteristics were determined by using a Monte Carlo test with 999 permutations. The relative contribution of each environmental variable to the community variation was calculated using rdacca.hp package [39]. All analyses for this paper were completed via R 3.6.0 [40], and *p* < 0.05 was used as the criterion for significant differences.

## 3. Results

### 3.1. Chemical Properties of Soil

Compared with MB, the SOM in JC was reduced by bamboo expansion, decreasing by 19.7% and 31.2% in the 0−10 cm and 10−20 cm soil layers, respectively, although the differences were not significant (Table 1). The soil TN concentration was consistent among the three stands, whereas the soil TP concentration in the 10−20 cm layer of BC was significantly higher than that of JC and MB (*p* < 0.05, Table 1). With bamboo expansion, the AK concentration showed an increasing tendency in the 0−10 cm soil layer, while the pH value had increasing tendencies in both layers (Table 1). Overall, the chemical properties of soil did not change significantly during bamboo expansion.

### 3.2. Overall Pyrosequencing Information

**Figure 1:**
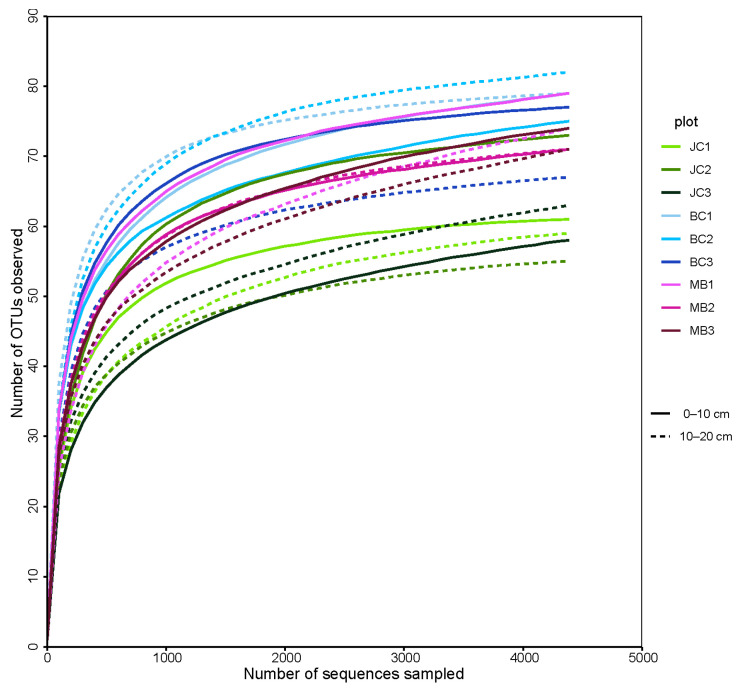
The rarefaction curves for OTUs-sequences. Note: JC, BC, and MB indicate Japanese cedar, bamboo-cedar mixed, and moso bamboo forest, respectively.

The number of sequences detected in JC, BC, and MB was 49194 (ranging from 7720 to 8731 per plot), 48856 (ranging from 6689 to 9019 per plot), and 46935 (ranging from 6650 to 8458 per plot), respectively. The rarefaction curves for the observed AMF OTU number and sequences sampled showed that the threshold for detecting the vast majority of OTUs was approximately 1500 (Figure 1).

### 3.3. The Richness and α Diversity of AMF

The bamboo expansion into Japanese cedar forest significantly increased the richness of AMF (*p* < 0.05, Table 2). The OTU number increased from 63 in JC to 77 in BC and 74 in MB in the 0−10 cm layer, from 59 in JC to 76 in BC and 72 in MB in the 10−20 cm layer (Table 2). The Shannon–Wiener, Simpson and Evenness indexes were significantly different among stands (*p* < 0.05). Specifically, these indexes in BC were significantly higher than those in JC and MB, and there was no difference in these indexes between JC and MB. Simultaneously, neither the richness nor diversity indexes of AMF changed with soil depth (*p* > 0.05, Table 2). Briefly, the *α* diversity of AMF was not changed after JC was succeeded by MB.

### 3.4. The Composition of AMF Community

**Figure 2:**
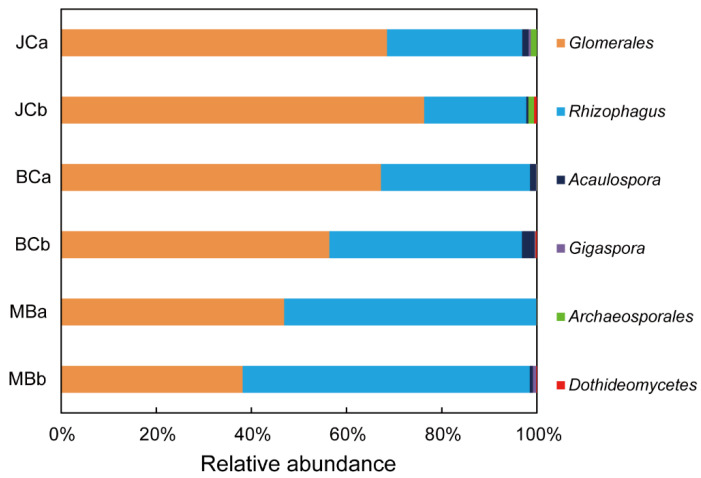
The soil AMF community composition.

The AMF assemblage in JC was altered by bamboo expansion (Figure 2). Specifically, *Glomerales* was the most abundant taxon in JC, with a relative abundance of 72.4%, which decreased to 61.8% and 42.5% in BC and MB, respectively (*p* = 0.321, *p* = 0.002). The relative abundance of *Rhizophagus*, the second most abundant taxon, increased from 24.9% in JC to 35.9% and 56.7% in BC and MB, respectively (*p* = 0.262, *p* < 0.001). The relative abundance of the remaining AMF taxa (*Acaulospora*, *Gigaspora*, *Archaeosporales*, *Dothideomycetes*) was low and did not vary with bamboo expansion. The relative abundance of all AMF taxa did not vary with soil depth (*p* > 0.05).

**Figure 3:**
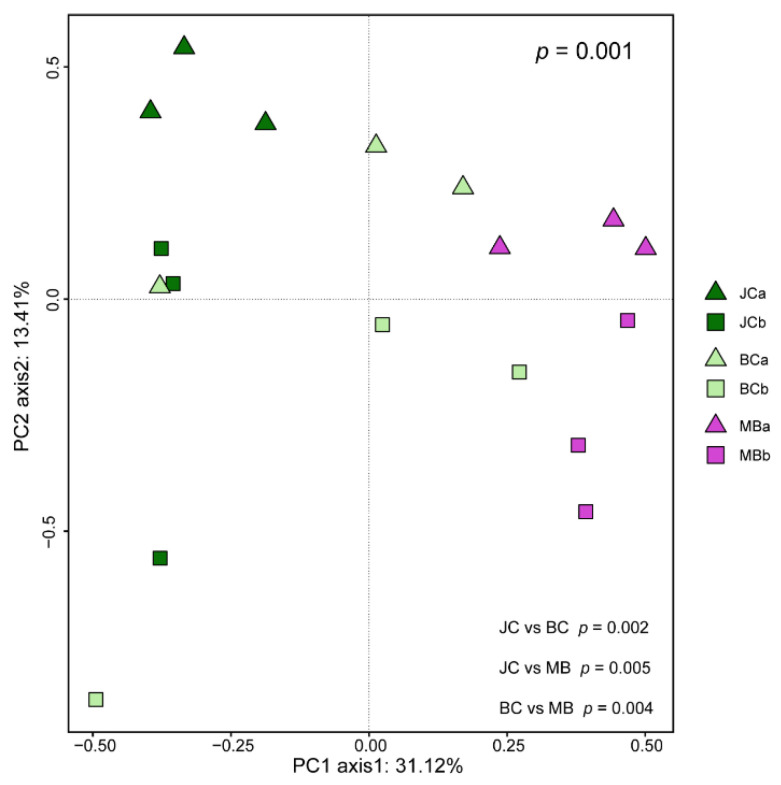
Principal component analysis (PCA) of the AMF community (represented by OTUs). Note: JCa and JCb are 0−10 cm soil layer and 10−20 cm soil layer of Japanese cedar, respectively; BCa and BCb are 0−10 cm soil layer and 10−20 cm soil layer of bamboo–cedar mixed, respectively; MBa and MBb are 0−10 cm soil layer and 10−20 cm soil layer of moso bamboo, respectively.

**Figure 4:**
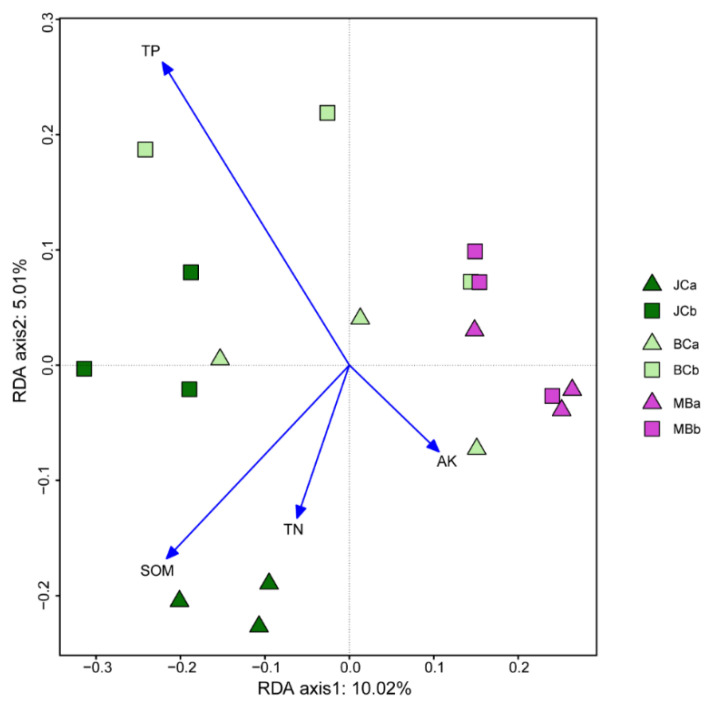
Redundancy analysis (RDA) of the AMF community (represented by OTUs). Note: JCa and JCb are 0−10 cm soil layer and 10−20 cm soil layer of Japanese cedar, respectively; BCa and BCb are 0−10 cm soil layer and 10−20 cm soil layer of bamboo–cedar mixed, respectively; MBa and MBb are 0−10 cm soil layer and 10−20 cm soil layer of moso bamboo, respectively. SOM, TN, TP, AP, and AK indicate soil organic matter, total nitrogen total phosphorus, available phosphorus, and available potassium, respectively.

Principal component analysis (PCA) based OTUs showed the distribution of soil AMF communities in constraint axes (Figure 3). The first and second principal components (*x*-axis and *y*-axis) explained 31.1% and 13.4% of the total variation in the AMF communities, respectively. In addition, AMF communities differed significantly among stands (*p* = 0.001, Figure 3), with significant differences between any two stands (*p* < 0.05, Figure 3). Redundancy analysis (RDA) showed that the soil chemical characteristics (SOM, TP, TN, and AK) accounted for 19.2% of the AMF community variation across stands (Table 3). The relative contributions of SOM, TP, TN, and AK were 44.4%, 37.2%, 18.3%, and 0.1%, respectively, with the first three factors being significant (Table 3). The first and second axis accounted for 10.0% and 5.0% of the AMF community variation, respectively; the former axis separated JC from MB, with BC being intermediate (Figure 4). Anyway, both univariate and multivariate statistical results showed that bamboo expansion altered the composition of AMF community.

## 4. Discussion

The expansion of moso bamboo into adjacent Japanese cedar changed the vegetation directly. Hence, we hypothesized that the AMF community would be altered by bamboo expansion. Our results indicated that the bamboo expansion did change the composition but not the diversity of AMF community. The relative contribution of soil chemical characteristics to AMF variation was low, and vegetation transformation was the main potential contributor to this variation.

Bamboo expansion reduced the quality and production of litter, and hence affected soil chemical characteristics [3,41]. Our results showed that the expansion of moso bamboo into Japanese cedar reduced the SOM, which is in line with previous studies focusing on bamboo expansion into coniferous and broadleaved forests [16], and Japanese cedar [42,43]. Bamboo expansion reduced the carbon content of litter, especially in coniferous forests [16]. Moreover, the herbaceous litter of bamboo decomposes easily, accelerating the eluviation of SOM, whereas the decomposition of Japanese cedar litter is extremely slow [44]. Hence, the expansion of moso bamboo into Japanese cedar reduced SOM. The pH value of the soil in this study tended to increase during bamboo expansion. The findings of a recent study, exploring the effects of bamboo expansion on soil pH across subtropical forests in China, supported our results [45]. The AMF community is also affected by soil chemical characteristics [46]. Similarly, Li et al. [18] reported that the changes in the soil fungal community during bamboo expansion into broadleaved areas were linked to changes in the soil’s chemical characteristics. Our research further identified that soil change can also be an avenue for bamboo expansion to change the AMF community in Japanese cedar.

Plant invasion influences the microbial community, and the positive or negative feedback of microorganism–plant interactions in turn affects the invasion [47]. Bamboo expansion is a form of plant invasion and has been shown to alter the fungal community [18], the AMF community [27], and the soil autotrophic bacterial communities in broadleaved forests [48]. In Japanese cedar forests, in addition to altering the bacterial communities [49] and microbial activities and community structure [43], our research further revealed that bamboo expansion increased the richness of AMF. AMF are strictly dependent on the host plant for their carbon supply [19]. Moso bamboo invests more in belowground growth [50], and its fine root biomass and specific root length were also significantly higher than those of the broadleaved trees [51], both of which can promote the mycorrhizal symbiosis in moso bamboo. Hence, the AMF richness in MB and BC was significantly higher than that in JC. The increased richness of AMF, in turn, enhanced the competitiveness of bamboo by facilitating nutrient absorption and stress tolerance, and then accelerated expansion.

Alpha diversity reflects not only the relative abundances of species and the differences between them, but also the function of the community due to the correlation between function and community structure [52]. We found that the α diversity of AMF was similar between JC and MB, indicating the increase in AMF species was relatively proportionate, and that BC had significantly higher diversity. Qin et al. [27] reported that the diversity of AMF was significantly higher in the ecotone formed by bamboo expansion into a broadleaved forest. Our research extended this pattern in the context of bamboo expansion into Japanese cedar. Bamboo expansion generally decreased biodiversity, including plants, animals, soil mesofauna, and microorganisms, but the literature shows inconsistent patterns of diversity change [4,53]. This variation could be attributed to the complex scenario of bamboo expansion. The types of replaced forest regulate the plant diversity variation during the expansion. The plant diversity of a monoculture plantation is consistent after being replaced by moso bamboo, whereas the diversity of mixed forest will dramatically decrease after being replaced by moso bamboo. The higher diversity of producers contributes to the higher diversity of consumers and microorganisms [54]. Although AMF–plant associations are not always species-specific, host plants favor certain AMF [55]. Moso bamboo and Japanese cedar have preferences for specific AMF; hence, the ecotone (BC) has higher AMF diversity than that of JC and MB. Similarly, Qin et al. [27] also found that the AMF diversity of a monoculture forest (moso bamboo) was lower than that of a mixed broadleaved forest, which supports our viewpoint. The diversity of AMF in JC increased during the process of bamboo expansion and then decreased after being completely replaced by bamboo. Both our and Qin et al.’s [27] results provide evidence to support the driver hypothesis that plants drive the interdependence between AMF and plants [56]. Considering the low relative contribution (19.2%) of soil chemical characteristics to the variation of AMF community across forest types, we speculated that the AMF community was affected largely by host plant change.

Regarding the specific AMF taxa, *Glomerales* (including *Rhizophagus*) was the dominant taxon in each stand. Previous research identifying AMF taxa by both spore [57] and high-throughput sequencing [58] demonstrated that *Glomerales* was the dominant taxon. *Glomus*, belonging to *Glomerales*, not only has a high capacity for survival and reproduction [59], but also has strong resistance to environmental stress [60]. The significant increase in the relative abundance of *Rhizophagus* during the transformation from JC to MB indicated moso bamboo preference for this genus, or at least some species of this genus. Root exudates differ even within species, and their effects on AMF have been clearly documented [26]. Simultaneously, the N form used by moso bamboo and Japanese cedar is also different, the former shows a preference for ammonium N [61], whereas the latter shows a preference for nitrate N [62]. Accordingly, this preference can be partly attributed to the variation in root exudates and nutrient utilization strategies. Nevertheless, the detailed chemical components and their influences on AMF, and the specific species and their functions in nutrient utilization, should be determined by further study.

## 5. Conclusions

The expansion of moso bamboo into monoculture Japanese cedar could affect AMF. Our results showed that bamboo expansion altered the community composition but not the *α* diversity of AMF. The relative abundance of *Glomerales* decreased, whereas that of *Rhizophagus* increased during bamboo expansion. Meanwhile, the richness and *α* diversity of AMF in the ecotone (bamboo-cedar mixed forest) were higher than those in the monoculture forest. In addition, the alteration of the AMF community was mainly driven by vegetation transformation. Putting these results into the current study context, we emphasize that bamboo expansion has effects on AMF community composition, but the effects on AMF diversity depend on the type of invaded forest. Nevertheless, the following question deserves to be addressed: Do the changes in AMF fungal community structure still maintain functionality?

## Figures and Tables

**Table 1:** The soil chemical characteristics of the three stands.

Stands	Soil Depth (cm)	SOM (g·kg^−1^)	TN (g·kg^−1^)	TP (mg·kg^−1^)	AK (mg·kg^−1^)	pH Value
JC	0~10	98.06 ± 13.50 a	3.32 ± 0.47 a	184.66 ± 19.29 c	71.65 ± 13.39 ab	4.66 ± 0.07 a
	10~20	78.68 ± 20.94 a	2.54 ± 0.59 a	298.10 ± 35.00 b	48.05 ± 12.22 b	4.73 ± 0.14 a
BC	0~10	78.37 ± 16.70 a	3.24 ± 0.63 a	258.92 ± 23.52 bc	84.41 ± 8.58 ab	4.70 ± 0.05 a
	10~20	54.73 ± 11.63 a	2.32 ± 0.44 a	399.92 ± 30.42 a	42.95 ± 3.36 b	4.76 ± 0.03 a
MB	0~10	80.19 ± 4.23 a	3.47 ± 0.15 a	194.47 ± 20.29 c	116.93 ± 16.58 a	4.78 ± 0.08 a
	10~20	54.10 ± 0.06 a	2.15 ± 0.06 a	233.13 ± 13.84 bc	49.64 ± 11.53 b	4.90 ± 0.07 a

Note: Data are means ± SE (*n* = 3). Different lowercase letters indicate significant differences among groups (*p* < 0.05) according to the LSD multicomparison. JC, BC, and MB indicate Japanese cedar forest, bamboo-cedar mixed forest and moso bamboo forest, respectively. SOM, TN, TP, AP, and AK indicate soil organic matter, total nitrogen total phosphorus, available phosphorus, available potassium accordingly.

**Table 2:** The richness and *α* diversity of the soil AMF community.

Stands	Soil Depth (cm)	Richness		Shannon-Wiener		Simpson		Evenness	
JC	0~10	63.00 ± 4.58 bc	B	2.84 ± 0.14 b	B	0.891 ± 0.023 a	B	0.687 ± 0.023 b	B
	10~20	59.00 ± 2.31 c		2.80 ± 0.07 b		0.894 ± 0.006 a		0.686 ± 0.013 b	
BC	0~10	77.00 ± 1.15 a	A	3.40 ± 0.02 a	A	0.943 ± 0.006 a	A	0.782 ± 0.004 a	A
	10~20	76.00 ± 4.58 a		3.40 ± 0.14 a		0.940 ± 0.011 a		0.784 ± 0.027 a	
MB	0~10	74.33 ± 2.60 a	A	3.13 ± 0.14 ab	B	0.910 ± 0.018 a	B	0.725 ± 0.047 ab	B
	10~20	71.67 ± 0.67 ab		2.95 ± 0.13 b		0.891 ± 0.022 a		0.691 ± 0.031 b	

Note: Data are means ± SE (*n* = 3). Different lowercase letters indicate significant differences among groups according to the LSD multicomparison (*p* < 0.05). Different capital letters indicate significant differences among stand types according to the LSD multicomparison (*p* < 0.05). JC, BC, and MB indicate Japanese cedar forest, bamboo-cedar mixed forest and moso bamboo forest, respectively.

**Table 3:** The contributions of soil chemical properties to the variation of AMF community.

Model		Variable	*p* Value	Relative Contribution (%)
*p* value	**0.001**	**SOM**	**0.019**	44.43
		**TN**	**0.011**	18.33
Adj *R*^2^	0.192	**TP**	**0.016**	37.24
		AK	0.795	0.1

Note: Variables with significant regression coefficients in the model are shown in bold font (*p* < 0.05).

## Data Availability

The data presented in this study are available on request from the corresponding author.
